# Placental transporter localization and expression in the Human: the importance of species, sex, and gestational age differences^†^

**DOI:** 10.1093/biolre/iox012

**Published:** 2017-03-07

**Authors:** Natasha Walker, Panagiotis Filis, Ugo Soffientini, Michelle Bellingham, Peter J O’Shaughnessy, Paul A Fowler

**Affiliations:** 1 Institute of Medical Sciences, School of Medicine, Medical Sciences and Nutrition, University of Aberdeen, Aberdeen, UK; 2 Institute of Biodiversity, Animal Health and Comparative Medicine, College of Medical, Veterinary and Life Sciences, University of Glasgow, Glasgow, UK

**Keywords:** placenta, placental transport, human, ontogeny

## Abstract

The placenta is a critical organ during pregnancy, essential for the provision of an optimal intrauterine environment, with fetal survival, growth, and development relying on correct placental function. It must allow nutritional compounds and relevant hormones to pass into the fetal bloodstream and metabolic waste products to be cleared. It also acts as a semipermeable barrier to potentially harmful chemicals, both endogenous and exogenous. Transporter proteins allow for bidirectional transport and are found in the syncytiotrophoblast of the placenta and endothelium of fetal capillaries. The major transporter families in the human placenta are ATP-binding cassette (ABC) and solute carrier (SLC), and insufficiency of these transporters may lead to deleterious effects on the fetus. Transporter expression levels are gestation-dependent and this is of considerable clinical interest as levels of drug resistance may be altered from one trimester to the next. This highlights the importance of these transporters in mediating correct and timely transplacental passage of essential compounds but also for efflux of potentially toxic drugs and xenobiotics. We review the current literature on placental molecular transporters with respect to their localization and ontogeny, the influence of fetal sex, and the relevance of animal models. We conclude that a paucity of information exists, and further studies are required to unlock the enigma of this dynamic organ.

## Summary Sentence

This review summarises the existing knowledge of human placental molecular transporters (SLC and ABC superfamilies). We highlight areas where greater and more accurate knowledge is required and discuss weaknesses of animal models for the human.

## Introduction

The placenta is an important, ephemeral organ, and optimal function is essential for normal fetal development and a successful pregnancy. It is well known that the placenta acts as a conduit to supply nutrients and remove metabolic waste from the fetus. The placenta also synthesizes and secretes hormones such as estrogens, progestogens, human chorionic gonadotrophin, and human placental lactogen which are essential for the establishment and maintenance of the pregnancy. Vitally, it acts as a gate keeper, regulating the flux of drugs and xenobiotic compounds in and out of the fetal compartment. It is the mother that ensures such compounds are present in the fetoplacental environment and their availability greatly depends on maternal lifestyle factors including but not limited to environmental chemicals (e.g., endocrine disruptors and environmental toxins), pharmaceuticals, illicit/recreational drugs, tobacco smoke constituents, and alcohol. It is important to note that such exposures may harm the fetus not only directly but also indirectly by perturbing placental function itself, impacting on fetal health without ever reaching the fetal compartment.

It is increasingly recognized that after parturition the placenta is not simply a waste by-product of pregnancy, but rather an organ that carries a fingerprint about important pregnancy parameters (e.g., pregnancy progression, substance exposure, fetal metabolism). This “placental fingerprint”, unique to every pregnancy, potentially encompasses biomarkers that can be used to make predictions about the offspring's health. For example, the shape and size of the term placenta can predict blood pressure in childhood, with reduced placentation (smaller placentas) resulting in higher systolic blood pressure due to undernutrition [1]. Pregnancy disorders such as intrauterine growth restriction, pre-eclampsia, and even miscarriage remain relatively poorly understood but their etiology is likely to include elements of placental dysfunction [2, 3]. In addition to perinatal adversities, the “Barker Hypothesis” (also known as “fetal origins of adult disease hypothesis”) postulates that long-term health problems can manifest due to perturbed fetal programming [4]. Since placental function is a determinant of both fetal development and postnatal health, a better understanding of placental molecular processes that define the placental fingerprint (i.e., which genetic/molecular/gross morphology markers the placenta has at term) will improve our understanding of these complications and will aid the detection of susceptible individuals.

The placenta is a highly dynamic structure throughout its 9-month lifespan, which not only grows in proportion to the fetus but also changes both structurally and morphologically. The demand for increased feto-maternal communication during gestation dictates branching and development of placental mesenchymal villi into terminal villi [5] thereby increasing the transport epithelial interface. In the later stages of the first trimester, the placenta is fully functional and serves the ever-increasing requirements of the fetus. Global gene expression is adjusted constantly and genes relating to metabolism are highly expressed in the first trimester whereas signal transduction pathways are more prominent in term placenta [6]. Due to a dynamic molecular environment, the profile of transcripts encoding molecular transporters adapts throughout the pregnancy as discussed below.

### Transplacental transport

**Figure 1.**
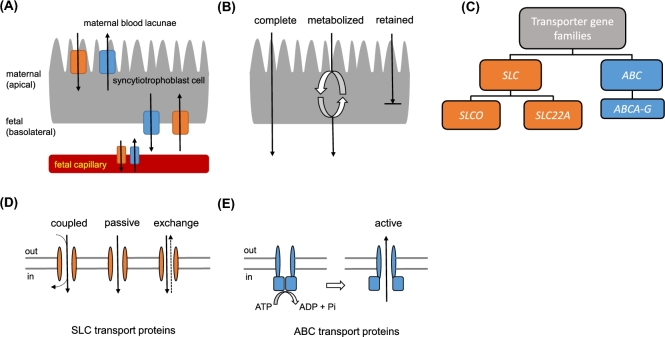
Transporting epithelium of the placenta and molecular transporter function. A molecule must be transported (or diffuse) through the syncytiotrophoblast and endothelium of the fetal capillary to access the fetal compartment. (A) Molecular transporter proteins are positioned in the maternal-facing apical and fetal-facing basolateral membranes and in the endothelium of fetal capillaries. (B) Possible pathways across the syncytiotrophoblast. A molecule can use transporter proteins, passive diffusion, or pinocytosis to cross the syncytiotrophoblast membranes. The molecule can be transported intact or may be metabolized first. The syncytiotrophoblast also retains certain molecules for its own use. (C) Superfamilies of genes coding for molecular transporters found in the placenta. SLC can be divided into gene subfamilies *SLCO* and *SLC22A*. ABC consist of seven gene subfamilies: *ABCA, ABCB, ABCC, ABCD, ABCE, ABCF, ABCG* although *ABCE* and *ABCF* are unlikely to have transporter function. A list of corresponding proteins can be found in Table 1. (D) Mechanism of transport for SLC proteins. Compounds can enter/exit the cell coupled to another molecule, passively down its concentration gradient or in exchange for another molecule. SLC transporters can be bidirectional although they mainly allow for placental uptake of molecules. (E) Mechanism of transport for ABC proteins. ABC transporters actively efflux compounds against their concentration gradient. ATP hydrolysis is required for a conformational change of the transporter protein to allow uptake and expulsion of the substrate from the cell.

The placenta is the first fetal organ to be fully developed during pregnancy. At around 7–12 days postconception, the epithelium of the placenta, the syncytiotrophoblast, invades the endometrium [7]. The syncytiotrophoblast is the transporting epithelium of the human placenta and its formation initiates an active border between mother and fetus, enabling molecular exchanges between the two. It is composed of an apical brush border which is bathed in a maternal blood lacunae and a basolateral membrane which encircles branches of the fetal capillary tree (Figure 1A). For a molecule to translocate from mother to fetus and vice versa, it must cross the syncytiotrophoblast, where it may be metabolized (Figure 1B), and the endothelium of the fetal capillary. Transplacental transport involves passive diffusion, pinocytosis, and facilitated and active transport via molecular transporter proteins which also support the barrier function of the placenta. Suboptimal placental performance may, therefore, result by disruption of the mechanisms that control those three modes of molecular transportation. Active transport via molecular transport proteins controls the flux of a wide variety of compounds and can be tightly regulated by controlling expression, localization, and activity of these transport proteins. For instance, polarized localization of transporter proteins in the syncytiotrophoblast and the endothelium of the fetal capillary can control directionality of the molecular flux between the mother and the fetus.

Solute carriers (SLC) and ATP-binding cassette proteins (ABC) are two major superfamilies of mammalian transporters. A schematic diagram of the *SLC* and *ABC* gene families is shown in Figure 1C. SLC transporters are capable of bidirectional transport but mainly deal with influx of substrates (Figure 1D) and can be further classified into subfamilies SLCO and SLC22A. ABC transporters are involved in substrate efflux (Figure 1E) and include subcategories A–F although it is considered unlikely that E and F have transporter function as they do not encode transmembrane domains [8]. Both superfamilies are involved in drug transport with ABC proteins more likely to govern fetal protection due to their active efflux properties, which move harmful xenobiotics/drugs away from the fetal circulation. Although many SLC and ABC transporters have been found in the placenta, very few have been characterized in terms of quantity, localization, gestational expression, degree of functionality, and substrate preference within the placenta. Membrane localization of some transporters has been well defined in the human although there is disagreement in the literature about how expression changes during gestation (Table 1).

**Table 1.** ABC and SLC transporters known to show expression in the human placenta.

Transporter	Syncytiotrophoblast localization	Substrates/function	References
ABCA1	Apical	Cholesterol, phospholipids	[99–101]
ABCB1	Apical	Drug resistance (antibiotics, antiemetic, cardiac drugs, HIV protease inhibitors)	[15, 16, 28]
ABCB4	Basolateral	Bile acids	[24, 38]
ABCC1	Apical	Folate	[28, 32]
ABCC2	Apical	Folate, bilirubin, role in chemoprotection and detoxification	[32, 34, 102, 103]
ABCC3	Apical	Bilirubin	[102]
ABCC4	Apical	Conjugated bile acids	[102]
ABCC5	Basolateral	Cyclic nucleotides	[35]
ABCC7	Apical	Chloride transport	[104]
ABCG1	Basolateral	Cholesterol, phospholipids	[100]
ABCG2	Apical	Drug resistance	[14, 105]
SLCO2A1	Unknown	Prostaglandin	[48]
SLCO2B1	Basolateral	Sulfated steroids (DHEAS), glutamate	[49, 50]
SLCO4A1	Apical	Thyroid hormones	[51]
SLCO1A2	Apical	Thyroid hormones, bile acids, conjugated steroid hormones	[46, 47]
SLCO1B1	Apical	Bile acids	[24]
SLCO3A1	Apical	Bile acids	[24]
SLC22A6	Unknown	Drug xenobiotic eliminator	[56]
SLC22A11	Basolateral	Sulfated steroids (DHEAS)	[57, 58]
SLC22A3	Basolateral	Cationic compounds	[52]
SLC22A5	Apical	Lactate, folate, carnitine	[53–55]
SLC22A4	Apical	Lactate, folate	[59]

Over- or underexpression of transporters would be expected to have effects on delivery and clearance of molecules at the maternal–fetal barrier impacting on fetal growth and health. For example, increased molecular transport, as seen in some diabetic mothers [9], could lead to an abnormally large fetus increasing the risk of metabolic syndromes later in life [10]. Conversely, malfunction or underexpression of transporters, or even competition for binding sites between nutrients and xenobiotics [11], could cause suboptimal delivery of nutrients to the fetus and may lead to growth restriction and concomitant health-associated risks for the neonate including cardiovascular risk [12] and type 2 diabetes [13]. These examples highlight the importance of appropriate and timely placental regulation of transporter expression. Recognizing transporter expression dynamics across fetal parameters such as gestation, sex, and maternal lifestyle has the potential to allow us to assess levels of protection offered by the placenta, and to identify instances of increased susceptibility, especially during critical periods such as organogenesis.

This review will discuss molecular transport proteins of the SLC and ABC gene superfamilies in the placenta, their localization and their expression profile across the trimesters. The review concentrates on data derived from studies using human tissue or human-derived cell lines due to the vast interspecies differences in placental structure and function, as discussed below.

## ABC superfamily

**Figure 2.**
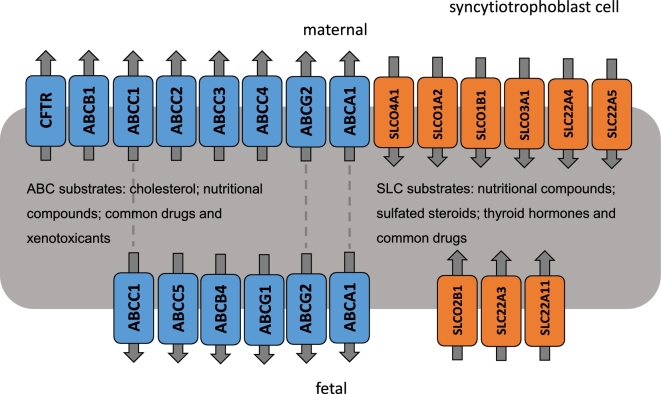
Molecular transporter transcripts with confirmed syncytiotrophoblast location in the human placenta and their direction of transport. SLC (orange) and ABC (blue) are found in both apical (maternal-facing) and basolateral (fetal-facing) membranes. As seen by the schematic, understanding of complete transplacental routes for compound movement is incomplete. Grey dashed line indicates the transporter has been found to be expressed on both membranes. It is likely that many molecules must use a combination of transporters to cross the membrane and as many transporters have common substrates, this is entirely possible. Substrates mentioned here are generalized substrates of the superfamilies.

ABC proteins actively transport physiologically relevant compounds such as inorganic ions, glucose, amino acids, metal ions, cholesterol, and phospholipids. In addition to this, they have a protective function by acting as efflux pumps to move pharmaceuticals and xenotoxicants away from the fetus and into the maternal circulation and they are the most widely studied transporter family within the placenta. Over 50 ABC transporters have been identified in humans and three subfamilies are involved in drug/xenobiotic efflux: ABCB, ABCC, and ABCG proteins. Many of those transporters have been found in the human placenta at the transcript and/or protein level (Figure 2).

### ABCB transporters

The ABCB subfamily is the most extensively studied of the ABC transporters due to its involvement in drug resistance. *ABCB1* and its corresponding protein product (also known as P-glycoprotein (P-gp)) are of particular interest in the placenta as they are highly abundant [14]. So much so that ABCB1 is also known as the “ABC placental transporter” and was the first discovered to offer fetal protection against toxicity. The apical membrane of the syncytiotrophoblast is enriched with ABCB1 [15], which supports its function as a drug extrusion pump from the placenta back toward the maternal circulation. This avoids drug build-up in the placenta and escape toward the fetus. Known substrates of ABCB1 include cytotoxic, antiemetic and cardiac drugs, antibiotics, and HIV protease inhibitors [16]. It has been reported that ABCB1 is significantly upregulated (30%) in placentas that are exposed to methadone (with or without heroin and cocaine), a known substrate of this transporter [17]. Many studies have reported that mRNA and protein levels decrease significantly from the first to third trimesters in the human placenta [18–21]. A possible explanation for this marked reduction in human placental ABCB1 could be the early need for retrograde transport of drugs/chemicals during sensitive critical periods of organogenesis.

ABCB4 is involved in bile acid transport and is believed to show drug interaction profiles similar to those of ABCB1 [22]. Its basal location in syncytiotrophoblast cells [23] suggests that it moves molecules out of the placenta, thereby potentially protecting the placenta from chemotherapeutic drugs. ABCB4 transcript expression has been reported to be fourfold higher in the third trimester compared to first [24] although why expression increases towards term is unclear. The importance of this transporter is seen in cases where the mother carries an ABCB4 mutation, causing intrahepatic cholestasis of pregnancy and impaired transplacental bile acid transport which can sometimes result in fetal death [25].

### ABCC transporters

The ABCC gene subfamily currently consists of seven multidrug resistance proteins [26] which transport conjugated and unconjugated organic ions and act as drug efflux pumps. Four of these proteins have been well characterized in the human placenta and ABCC1 is more abundant in placenta than in liver or kidney [27]. ABCC transporters are localized on opposing sides of the syncytium which may give us insight to their physiological roles. ABCC2 and ABCC3 can be found on the apical membrane, directing solutes toward the mother whilst ABCC1 and ABCC5 are localized basolaterally and would be expected to move molecules toward the fetus. This means that the latter two transporters would not be expected to play a role in reducing fetal exposure unless there was a change in expression levels. There is also evidence of ABCC1 localization on the apical membrane and high expression levels in the fetal capillary endothelium of both ABCC1 and ABCC3 [28]. These transporters also help in the clearance of biliary substances from fetal liver to maternal circulation, which is essential to avoid harmful build-up [29].

Another important substrate of the ABCC group is folate which is so essential to avoid neural tube defects in the fetus that women are advised to take extra supplementation during the periconceptual period [30]. ABCC1 and ABCC2 assist in folate transport across the maternal–fetal barrier. Currently, studies of ABCC1 expression levels during development are contradictory with levels reported to either increase [31] or decrease [32] with gestational age. If the age-related increase is correct, it could indicate increasing demand during a period of accelerated fetal growth and aligns with clinical guidelines for folic acid supplementation during the early stages of pregnancy. Conversely, if the reported decrease in ABCC1 is correct, this could suggest that this transporter is important in the earlier stages of pregnancy, during establishment of the placenta, and that folate is preferentially moved via other transporters later on. It should be noted that, either way, the fetus has chronic requirements for folic acid as there is evidence that it is required in other processes essential for a progressing pregnancy (e.g., angiogenesis) [33]. Nevertheless, the contradiction between the studies of [31] and [32] needs to be resolved in order to understand the role of ABCC1 in human placental function. ABCC2 is involved in the excretion of conjugated bilirubin and drugs, including anticancer pharmaceuticals [26]. ABCC2 has been shown by two groups to increase in the placenta during pregnancy with an associated increase in bilirubin expulsion, [32, 34]. ABCC5 transports cyclic nucleotides and is in a fetal-facing position on the syncytiotrophoblast; thus, it is unlikely to have a role in fetal protection. ABCC5 and its transcript, *ABCC5*, decrease across gestation [35].

### ABCG transporters

Transporters in this family with identified functions in the placenta include ABCG1, involved in cholesterol transport, and ABCG2 (more commonly known as breast cancer resistance protein (BCRP)) involved with drug resistance. The placenta expresses higher levels of ABCG2 than any other organ [36]. ABCG2 has many exogenous and endogenous substrates and shares some of these with ABCB1. ABCG2 is localized to the apical membrane of syncytiotrophoblasts and the endothelium of fetal capillaries [37]. It has been hypothesized that ABCG2 is the placental “survival factor” during placental maturation and offers protection from cytokine-induced apoptosis [38]. ABCG2 may also be susceptible to maternal bisphenol A and para-nonylphenol exposure both of which are associated with downregulation of the protein [39].

Expression patterns of ABCG2 during pregnancy remain uncertain due to discrepancies in the literature [18, 21, 39–42]. More studies are needed to single out the functionally important reasons for these differences which may simply reflect cohort variation or may be due to differences in methodology. Variations in the normalizing genes used may also add to discrepancies in the literature as there are global, sex-dependent gene changes in the placenta across pregnancy and use of a stable combination of housekeeping genes is essential for the production of meaningful data [43]. Studies which correctly account for fetal sex, cover as many gestational weeks as possible and establish the most stable method of normalization are needed.

### SLC superfamily

This superfamily is extremely large with 55 families and 300 members (SLC1-20 SLC22-47 then SLCO2-6 and uncoupling protein UCP1-3) [44] which can be categorized according to Figure 1C. Their positions within the syncytiotrophoblast can be seen in Figure 2. SLCs have been identified in almost every human epithelium and were exhaustively reviewed by Roth et al. in 2012 [45]. Not all of these are found, or are functional, in the human placenta, and this section will only discuss transporters relevant to the in utero environment. Previous studies have reported placental expression of SLCOs (SLCO1A2 [46, 47], SLCO1B1 [24], SLCO3A1 [24], SLCO2A1 [48], SLCO2B1 [49, 50], SLCO4A1 [51], and low expression of SLCO6A1) and SLC22As (SLC22A3 [52], SLC22A5 [53–55], SLC22A6 [56], SLC22A11 [57, 58] with SLC22A1, SLC22A2, SLC22A4 [59] showing weak expression) (see Table 1). The placenta uses these ATP-independent uptake transporters to import hydrophilic or charged molecules into the cell, some of which are then used as hormonal substrates. As previously mentioned, they mainly orchestrate uptake of molecules into the cell using coupled, passive, and exchanger transport (Figure 1B). Substrates of this family are extensive but include many nutritional compounds such as amino acids, glucose and sugars, vitamins, fatty acids, inorganic and organic ions, oligopeptides, sulfated steroids, and thyroid hormones [45]. Common drugs, such as NSAIDS, antibiotics, diuretics, and anti-cancer drugs are transported by SLC transporters [60]. Members of the family can be specific or polyspecific to these substrates and are also involved in drug resistance. Since they transport a wide range of toxins, common drugs, and nutritional compounds, these transporters are extremely important in placental function. Exogenous compounds have also been shown to overcome the maternal–fetal barrier by acting as transport substrates of SLC membrane transporter proteins (i.e., xenobiotics [61]), suggesting that placental expression of those transporters can control the exposure of the fetus to potentially harmful compounds.

### Ontogeny of placental SLC transporters

Currently, data are limited with respect to the gestational expression of this family. The human-specific transporter SLC22A11 is found at high levels in kidney and at the basolateral membrane of the syncytiotrophoblast [57], where it imports sulfated dehydroepiandrosterone (DHEAS) into the placenta and away from the fetus [58] along with SLCO2B1, which has 10-fold lower affinity for DHEAS. This uptake is necessary for the placental synthesis of estrogens [62].

SLC22A4 and SLC22A5 are both expressed apically, although their gestational expression profiles are currently unknown. Both transporters import lactate and folate from the mother to the placenta, while SLC22A5 also uptakes L-arginine from the maternal side into the placenta and has been shown to import common drugs e.g., antibiotics and antidepressants [53]. SLC22A3 plays an important role in the transport of cationic compounds, but its full function within the placenta remains unknown although it may be placenta-specific as other tissues have very low expression [63]. SLC22A3 expression in pregnancy decreases between the first and third trimester [64].

Understanding the ontogeny of SLCO transporters is of intense interest due to their role in drug and essential hormone transport. Patel et al. [24] have reported that there are marked changes in SLCO transporter gene expression between the first and third trimester human placenta. Their data showed an 8-fold decrease in SLCO1A2 and a 17-fold decrease in SLCO3A1 (with no change in SLCO1B1 or SLCO4A1) during this period although the study was limited by a very small sample size (eight placentas at 9–12 weeks and six at term). Others have reported, in contrast, that SLCO1A2 increases toward term along with three other thyroid hormone transporters (SLC16A2, SLC16A10, SLC7A5) [65]. This latter study had a much larger sample size (n = 110) but the conflicting results highlight the need for further developmental studies on the placenta to be carried out. Prostaglandin (PG) transport is also mediated by SLCO proteins. SLCO2A1 is involved in the clearance of PG and was found to decrease with gestation [66].

## Limitations in placental transport studies

### Imperfect models for transport studies

Interspecies placental heterogeneity is considerable and consequently, a nonprimate (specifically non great ape) animal model that fully encompasses human placentation does not exist. This poses the question whether transporter expression and activity measured in an animal model has any relevance with respect to the human. Table 2 compares common animal models used for placental studies. There are obvious advantages to using animal models (in particular, it is just not possible to carry out many studies in the human for practical or ethical reasons), but there are also many drawbacks. For example, the syncytiotrophoblast in humans is not mimicked precisely by other animals, other than great apes, and therefore studies on transporter localization, or indeed time-dependent changes in expression, cannot be confidently extrapolated using nonhuman placental tissue. In addition, all animal models (and particularly rodents) have a shorter gestation than the human. In many cases, this means that they are not born at comparable stages of development so the dynamics of placental development is likely to be very different. Other differences are described in more detail below.

**Table 2.** Comparing animal placentas used for comparative studies against the human placenta.

Model	Days to term	Placental structure	Interhemal layers
Human	266–280	Discoid	3
Mouse/rat	20–22	Discoid	5
Guinea pig	59–72	Discoid	3
Sheep	147	Cotyledonary	6

Comparison of gestational timing indicates that offspring are born at different stages of development. Placenta structure is classified according to gross shape and points of maternal contact. Interhemal layers is the number of membranes that separate the maternal and fetal circulations thus representing number of membranes that molecules must either diffuse or be transported across. This highlights difficulties of using animal models to understand transport processes in human placenta. All from maternal to fetal direction. Human: syncytiotrophoblast; cytotrophoblast; fetal capillary endothelium. Mouse/rat: syncytiotrophoblast; additional syncytial layer; sinusoidal giant cell layer. Guinea pig: syncytiotrophoblast; basal lamina; fetal endothelium. Sheep: fetal maternal endothelium; maternal stroma; uterine epithelium; trophoblast layer; fetal stroma; endothelium of fetal capillary.

Along with primates and rodents, the human placenta has a discoid shape and hemochorial nature (maternal blood is in direct contact with syncytiotrophoblast). However, significant differences can be seen in the gross structural morphology between species (Table 2). There are marked differences between human and mouse placenta as previously reviewed [67] but only the dissimilarities affecting transporter studies will be discussed here. Rodents have an additional inverted yolk sac placenta that exists alongside the normal placenta throughout the pregnancy and is essential for proper development of the embryo [68]. It is impossible to separate this secondary structure which can lead to questionable findings in feto-maternal transfer studies. For some of the transport proteins, there are no orthologs between human and rodent and transporters can also be differentially localized, move dissimilar substrates, can be regulated differently, and are differentially responsive to inhibitors [69]. The syncytiotrophoblast in mice follows the same pattern as humans but encompasses an additional syncytial layer and sinusoidal giant cell layer. In the architectural sense, they have a labyrinthine exchange barrier whilst human have a villous barrier. Trophoblast invasion also differs between the species; rats and mice have a single cotyledon and three trophoblast cell layers whilst humans have a cluster of cotyledons and a single syncytial layer [70]. Functionally, human syncytiotrophoblast cells support both endocrine and transporting function but in the mouse these roles are geographically separated into a junctional and labyrinth zone, respectively. One similarity between the mouse and the human is the thinning of the syncytial membranes which increases diffusion capacity toward term, making the late gestation rodent placenta comparable to the human in nutrient diffusion conductance studies (e.g., oxygen) [71].

The guinea pig is also commonly used as a model of placental function and, in particular, for nutrient transport studies in order to understand intrauterine growth restriction [72]. Anatomically, guinea pigs have the complication of a subplacenta [73] (a convoluted area of trophoblasts which is separated from main placenta by a junctional zone) and yolk sac that persists until term. However, guinea pigs have a lengthier gestation than rats and mice and give birth to precocial young and so the placenta must support further developmental processes compared to other rodents and their altricial young [74]. The guinea pig feto-maternal exchange interface is also similar to human with a single layer (hemomonochorial) syncytiotrophoblast (Table 2), although the cytotrophoblast does not persist into the second half of gestation.

The sheep has a similar cotyledon to the human with comparable fetal vasculature [75] and maturity at birth although it is structurally different. The syncytiotrophoblast differs with more maternal layers being retained, which increases the number of layers between maternal and fetal vasculature (Table 2). There is also no maternal blood lacunae so villi do not bathe directly in maternal blood. The syncytiotrophoblast of the sheep is only perfused maternally as fetal cells do not invade the syncytium, although they are interdigitated to aid diffusion. The sheep placenta is not as well studied as, for example, the guinea pig, since amino acid transporters have not been mapped onto the syncytial layers as yet [76].

These species-dependent differences in syncytiotrophoblast development, structure, and function create considerable problems when relating animal studies to an understanding of transfer and transporter activity in the human placenta. There are also other problems to consider when extrapolating to the human. For example, human endocrinology of pregnancy is unique due to the high levels of estrogen generated by placental aromatization of fetal adrenal dehydroepiandrosterone. Other species lack significant placental aromatase (CYP19A1) [67], and this lack of CYP19A1 will also change the metabolic profile of the placenta affecting toxicity/toxicological studies. There are also molecular features which are unique to human, affecting trophoblast invasion (Siglec-6 and IMUP-2) [67].

Overall, the shortcomings of animal models described here demonstrate the need for researchers to use human-derived tissue in studies relating to placentation, common pregnancy disorders, and toxicology. In order to minimize potentially misleading extrapolations from animal models, there have been human models established at both ex vivo and in vitro levels. The term placental perfusion model, although only a representation of the final pregnancy stage, can be used, for example, to study transfer of solutes between mother and fetus [77]. This has been useful, especially, in drug transport studies [78] as it accurately represents the syncytiotrophoblast in a physiological environment. Non-differentiated cell lines have also been used to represent placental function at all weeks of gestation. The BeWo cell line, established in 1968 by Pattillo and Gey [79], is established for placental studies while the Jeg-3 and JAr cell lines are also popular. These three immortalized cell lines are all derived from human choriocarcinoma cells but they differ with respect to proliferation and differentiation. It is vital, therefore, to characterize transporter expression in each cell line in order to make informed decisions about which one to use when studying the placental barrier [27].

### Fetal age

There are, of course, a number of problems facing placental studies using human tissue. It is possible to collect tissues from normally progressing elective terminations (with a wide range of upper fetal age limits, from 12–20 weeks in different countries) and from term placentas but both require relevant ethical approvals which can be an arduous process. Even when tissues can be collected, what is generally missing are representative samples from the weeks between the latest elective termination date (after ∼20 weeks in the UK) and preterm births (∼38 weeks). Therefore, data from this period come largely from miscarriages where there are potential confounding factors with respect to fetal and/or placental normality. An unrepresentative age sample is a problem in studying the ontogeny of transporter expression as the transporter of interest may not be expressed during the timeframe leading to potentially erroneous conclusions.

### Fetal sex

A review by Clifton and Murphy [80] has stated that disregarding the sex of the fetus whilst studying the placenta is improper practice. While some studies do follow this advice [81, 82], most studies disregard the sex of the fetus and pool placental samples (e.g. [17–19, 23]). Trophoblast cells are derived from the embryo, however, and so they are sex-specific and recent studies have shown a marked difference in placental transcript levels between male and female fetuses [83]. There is also considerable evidence from other studies to suggest functional differences in the placentas from different sexes. As discussed above, influences of the intrauterine environment shape fetal programming and may lead to disease onset later in life [84]. Pertinently, it has been reported that the time to onset and severity of the resulting disorder differ between sexes [85]. Discrepancies in growth ratios between males and females were recognized 50 years ago [86], and altered fetal growth is known to be intimately associated with trophic delivery via the placenta [72]. This raises the question whether some of these sex differences could be explained by preferential transporter expression.

Currently, there are no data that show transporter expression being influenced by fetal sex in the human placenta. However, the endocrine environment of the fetus is sexually dimorphic and placental transporters are known to be regulated by hormones. ABCG2 expression, for example, has been reported to be altered by progesterone and estradiol exposure in the BeWo cell line [87]. Similarly, recent evidence has suggested that the adverse pregnancy outcomes in polycystic ovary syndrome could be caused by hormonal influence on amino acid transport across the placenta due to much higher circulating levels of androgens [88]. As the endocrine environment is established with respect to fetal sex from as early as 8 weeks of gestation, there may be sex-specific differential expression levels of transporters in both the fetus and placenta during pregnancy. A further complication with respect to the sex of the fetus has arisen from a study [89], which reported that housekeeping genes commonly used to normalize human placental qPCR studies differ in expression levels depending on fetal sex. This could either serve to accentuate or reduce sex-specific differences, thereby invalidating the study and highlighting the need to choose normalization strategies carefully.

### Medication in pregnancy

There is growing concern about the use of medication during pregnancy with evidence emerging for increasing use and prevalence of medication by pregnant women [90]. Medicating during pregnancy is often necessary to ameliorate acute or chronic illnesses of the mother or even to treat problems with the developing fetus, such as fetal arrhythmia or antiretroviral treatment if the mother is HIV positive. A major problem however is that, for good reasons, pregnant woman are normally excluded from drug trials, leading to a paucity of knowledge on correct dosage and usage of medications in expectant mothers [91]. There is a risk both of underdosing or overdosing and exposing the fetus to potential harm as many drugs are able to reach the fetal compartment [92]. There is also the potential for pregnancy-specific drug interactions as drugs crossing the syncytiotrophoblast could saturate transporters; indeed, certain antibiotics have been shown to block transporters [93]. Exogenous glucocorticoids are the preferred treatment for woman at risk of adverse pregnancy outcomes such as preterm birth and recurrent miscarriages. Glucocorticoids have been shown, however, to reduce amino acid transporter expression in vitro [94], and this altered transporter expression in the placenta could be the reason glucocorticoid use in pregnancy is linked to intrauterine growth restriction [95].

Understanding the developmental expression of the transporters may provide insights into both maternal and fetal medicating, especially with respect to when it is or is not safe to do so. ABC expression, for instance, changes throughout gestation and any decrease in protective drug efflux would aid in drug delivery to the fetal compartment. Nitrofurantoin, for example, is widely used to treat urinary tract infections and continued treatment is necessary, even in pregnancy, since a change in the bacterial environment heightens risk of preterm delivery. Nitrofurantoin is eliminated by ABCG2 [96] so any decrease in ABCG2 expression may harm the fetus, and knowing this time frame (currently a controversy in the literature) could inform medical professionals when to take precaution when prescribing. Analgesics are extensively used during pregnancy (e.g., to ease maternal discomfort) and are considered safe but recent data challenge this. A shortened anogenital distance has been observed, for example, in male fetuses exposed to paracetamol during weeks 8–14 of gestation [97]. The mechanism of this effect is not clear but paracetamol can decrease ABCG2 expression [98] suggesting that it may induce nonphysiological changes to transporter expression with downstream effects on placental function and fetal health—particularly as ABCG2 is one of the key players at the maternal–fetal interface.

In conclusion, there is a need to perform placental transporter studies using human tissue with consideration of fetal sex and with the largest age spectrum achievable. This would uncover more sex and/or time differences in transporter expression, data which the field is either lacking or unable to agree upon. It also may be of interest to study transporter expression in the fetal liver from the same pregnancy as it is the next major gate keeper to prevent fetal exposure, receiving 70% of the blood from the placenta. A better understanding of transporter regulation, both physiologically and pathologically, could shed light on molecular disposition within the placental and fetal compartments. Future studies following these criteria may demonstrate to what extent, and why, transporter expression is up/downregulated over the trimesters. It is also important to relate mRNA and protein levels to transporter activity within the human placenta, an area of research that is far from conclusive. Furthermore, convincing establishment of membrane localization of all identified transporters is required so that an accurate “map” of transport directions can be established.
